# Tension pneumocephalus: the neurosurgical emergency equivalent of tension pneumothorax

**DOI:** 10.1259/bjrcr.20150127

**Published:** 2016-05-08

**Authors:** John Julian Harvey, Simon Christopher Harvey, Antonio Belli

**Affiliations:** ^1^ Radiology Department, Auckland City Hospital, Auckland, New Zealand; ^2^ Radiology Department, Guy’s Hospital, London, UK; ^3^ NIHR Surgical Reconstruction and Microbiology Research Centre, Birmingham, UK; ^4^ Neurosurgery Department, The New Queen Elizabeth Hospital, Birmingham, UK

## Abstract

Tension pneumocephalus (TP) is the intracranial equivalent of tension pneumothorax. It is an unusual but life-threatening neurosurgical emergency, which has been described following head trauma, epidural injections or complicating neurological, spinal, craniofacial or sinus surgery. Unfortunately, the signs and symptoms of TP are non-specific and the diagnosis must be made by prompt recognition of the classic imaging signs of TP, allowing lifesaving emergency decompression. We present a trauma patient demonstrating the “Mount Fuji” sign on an unenhanced CT scan of the brain, which is reportedly specific for TP.

## Summary

Tension pneumocephalus (TP) is the intracranial equivalent of tension pneumothorax. It is an unusual but life-threatening neurosurgical emergency, which has been described following head trauma (associated with base of skull or sinus fractures), epidurual injections or complicating neurological, spinal, craniofacial or sinus surgery.^1–6^ Unfortunately, the signs and symptoms of TP are non-specific and the diagnosis must be made by prompt recognition of the classic imaging signs of TP, allowing lifesaving emergency decompression.^1,3–6^ We present a trauma patient demonstrating the “Mount Fuji” sign on an unenhanced CT scan of the brain, which is reportedly specific for TP.^5^

## Clinical presentation

An elderly male was found collapsed in his garden after a mechanical fall. On initial medical assessment by pre-hospital medical staff, his Glasgow coma scale (GCS) score was 14/15 (E4 V4 M6), which rapidly deteriorated to 11/15 (E3 V4 M4). He was transferred to the regional neurosurgical centre. Collateral history revealed that the patient had multiple co-morbidities: Type II diabetes mellitus, cirrhosis secondary to alcoholic liver disease and two previous myocardial infarcts.

**Figure 1.**
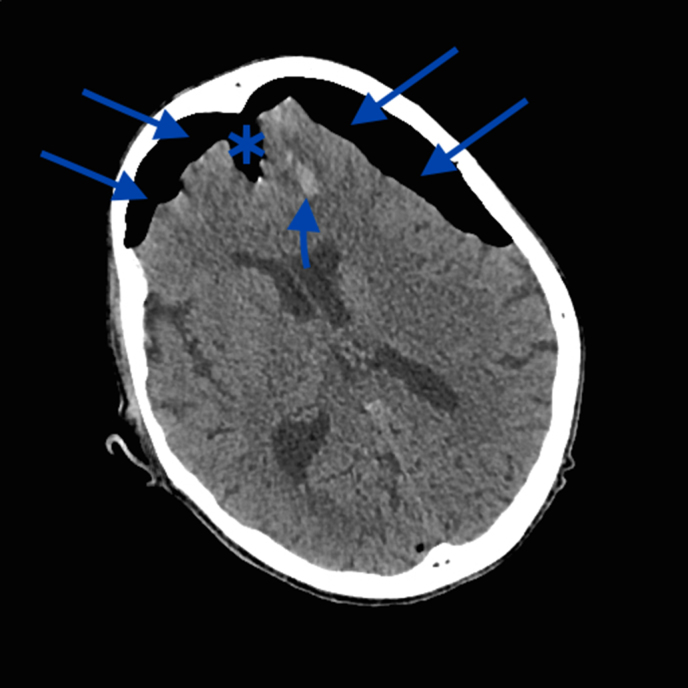
Unenhanced axial CT brain slice on brain windows demonstrating the “Mount Fuji” sign. There is a large volume of subdural air (long arrows) which is compressing and causing separation (star) of both frontal lobes away from the midline falx cerebri, pathognomic of tension pneumocephalus. A small heamorrhagic contusion (short arrow) is also seen.

**Figure 2.**
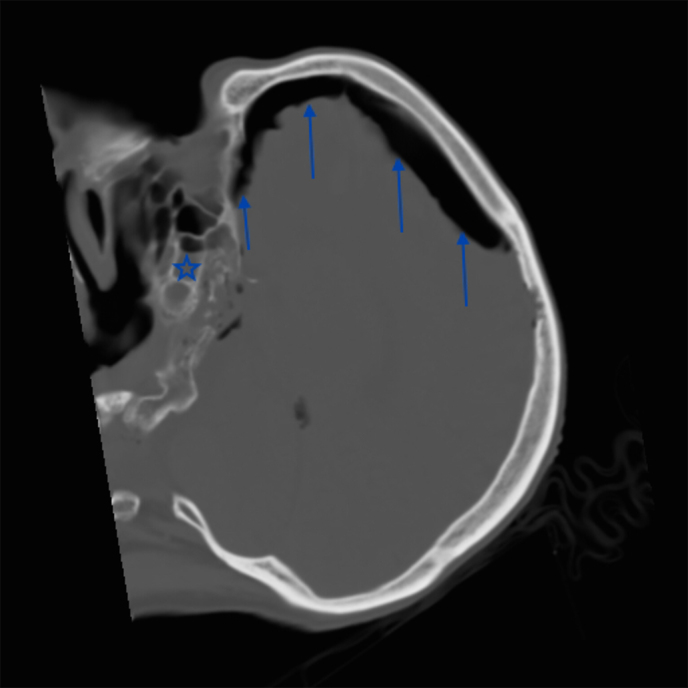
Sagittal CT brain image on bone windows demonstrating a large frontal subdural air collection (arrows) compressing the frontal lobe. The dependent air/fluid level in the sphenoid sinus raises the suspicion of an occult fracture (star).

On arrival at the hospital, the patient was maintaining his own airway, but remained drowsy. An urgent unenhanced CT scan of the brain was performed to exclude an intracranial haematoma, given the unclear history of blunt head trauma. The CT scan showed no extra-axial blood, but instead a large volume of intracranial air causing compression and separation of both frontal lobes away from the falx cerebri in the midline. This is called the “Mount Fuji” sign and is pathognomic of TP.^1,5^ Also noted were small haemorrhagic brain contusions and dependent fluid in the sphenoid sinus, raising the suspicion of a base of skull fracture, although no definite fracture could be identified (Figures 1 and 2). Urgent neurosurgical referral was made and the patient was prepared for emergency surgery.

The patient proceeded to the operating room where the neurosurgeon created bifrontal burr holes and durotomies. Although no audible air rush was heard, the patient’s consciousness improved rapidly to GCS 14/15 (E4 V4 M6) and he was returned to the ward to recover. Over the next 48 h, the patient made a steady recovery in the ward, but suffered an unexpected massive myocardial infarction from which he could not be resuscitated. All neurological observations had been stable until this point, so it was thought that this was unrelated to the TP, but rather owing to his significant past cardiac history. Several attempts to contact the patient’s next of kin and general practitioner to seek approval for case publication were unsuccessful. Accordingly, the case has been fully anonymized with regard to time, place and person.

## Discussion

TP can develop acutely (as in this case) or chronically over several months.^2,4^ The signs and symptoms of TP are non-specific, ranging from headache, hemiparalysis to coma, presumably reflecting increasing brain parenchymal compression.^3^ Fortunately, the wide availability and rapid access to CT head scanning allows rapid diagnosis of TP once the Mount Fuji sign is seen.^1,5^

TP is thought to arise from the one-way passage of air through a dural tear owing to increased nasopharyngeal pressure (*e.g.* with positive pressure ventilation or during coughing or sneezing), forming a “one way valve effect”, similar to that in tension pneumothorax.^3,4^ Alternatively, loss of the cerebrospinal fluid (CSF; traumatic or iatrogenic) may draw in air intracranially to replace the lost volume, the “inverted bottle effect”.^3,4^ Consequently, a subtle fracture or CSF leak should be actively sought clinically or radiologically when TP is seen on the CT scan. Extra-axial compression of brain parenchyma by the air in TP can lead to severe neurological defects and eventual fatal brainstem herniation, similar to that caused by other space occupying extra-axial masses, but with a more rapid time course.^2^

Patients with TP require urgent decompression and neurosurgical referral. Once recognized, the patients should be managed in a supine position (to minimize further CSF leakage) and given 100% supplemental oxygen to hasten removal of the intracranial nitrogen.^4^ In patients with existing burr holes or craniotomy, successful needle decompression (22 gauge) in the emergency department or under CT guidance has been described, prior to definitive neurosurgical repair of any cranial defect.^1,2^

## Learning points

1. Simple small volume pneumocephalus is common following head trauma or other conditions that breach the epidural space.
2. Accumulation of a large volume of constrained intracranial air can compress the brain parenchyma, causing TP, an unusual but rapidly life-threatening condition, similar to tension pneumothorax in the chest.
3. Signs and symptoms of TP are non-specific and varied: a high degree of clinical suspicion is needed.
4. The diagnosis of TP is easily made on a CT scan of the head by looking for the “Mount Fuji” sign, which differentiates it from simple pneumocephalus. The “Mount Fuji” sign is the extra-axial compression and parting of both frontal lobes by intracranial gas.
5. Once diagnosed, TP requires emergent referral to a neurosurgeon for decompression.

## Consent

The patient is deceased. The images are non-identifiable. JJH and AB (as first author and Head of Department, respectively) have completed and signed the patient consent waiver letter.
